# An Administrative Claims Model for Profiling Hospital 30-Day Mortality Rates for Pneumonia Patients

**DOI:** 10.1371/journal.pone.0017401

**Published:** 2011-04-12

**Authors:** Dale W. Bratzler, Sharon-Lise T. Normand, Yun Wang, Walter J. O'Donnell, Mark Metersky, Lein F. Han, Michael T. Rapp, Harlan M. Krumholz

**Affiliations:** 1 Oklahoma Foundation for Medical Quality, Oklahoma City, Oklahoma, United States of America; 2 Department of Health Care Policy, Harvard Medical School and Department of Biostatistics, Harvard School of Public Health, Boston, Massachusetts, United States of America; 3 Center for Outcomes Research and Evaluation, Yale-New Haven Hospital, and Section of Cardiovascular Medicine, Department of Internal Medicine, Yale University School of Medicine, New Haven, Connecticut, United States of America; 4 Pulmonary and Critical Care Unit, Massachusetts General Hospital and Harvard Medical School, Boston, Massachusetts, United States of America; 5 Division of Pulmonary and Critical Care Medicine, University of Connecticut School of Medicine, Farmington, Connecticut, United States of America; 6 Centers for Medicare and Medicaid Services, Baltimore, Maryland, United States of America; 7 Department of Emergency Medicine, George Washington University School of Medicine and Health Sciences, Washington, D.C., United States of America; 8 Robert Wood Johnson Clinical Scholars Program, Department of Internal Medicine, and Section of Health Policy and Administration, School of Public Health, Yale University School of Medicine, New Haven, Connecticut, United States of America; University of Giessen Lung Center, Germany

## Abstract

**Background:**

Outcome measures for patients hospitalized with pneumonia may complement process measures in characterizing quality of care. We sought to develop and validate a hierarchical regression model using Medicare claims data that produces hospital-level, risk-standardized 30-day mortality rates useful for public reporting for patients hospitalized with pneumonia.

**Methodology/Principal Findings:**

Retrospective study of fee-for-service Medicare beneficiaries age 66 years and older with a principal discharge diagnosis of pneumonia. Candidate risk-adjustment variables included patient demographics, administrative diagnosis codes from the index hospitalization, and all inpatient and outpatient encounters from the year before admission. The model derivation cohort included 224,608 pneumonia cases admitted to 4,664 hospitals in 2000, and validation cohorts included cases from each of years 1998–2003. We compared model-derived state-level standardized mortality estimates with medical record-derived state-level standardized mortality estimates using data from the Medicare National Pneumonia Project on 50,858 patients hospitalized from 1998–2001. The final model included 31 variables and had an area under the Receiver Operating Characteristic curve of 0.72. In each administrative claims validation cohort, model fit was similar to the derivation cohort. The distribution of standardized mortality rates among hospitals ranged from 13.0% to 23.7%, with 25^th^, 50^th^, and 75^th^ percentiles of 16.5%, 17.4%, and 18.3%, respectively. Comparing model-derived risk-standardized state mortality rates with medical record-derived estimates, the correlation coefficient was 0.86 (Standard Error = 0.032).

**Conclusions/Significance:**

An administrative claims-based model for profiling hospitals for pneumonia mortality performs consistently over several years and produces hospital estimates close to those using a medical record model.

## Introduction

Pneumonia, the second most common cause of hospitalization of the elderly, accounts for approximately 770,000 admissions annually among patients 65 years of age or older in the United States [1], [2]. Hospitalization rates for pneumonia have increased by 20% from 1988–1990 to 2000–2002 for patients aged 65 to 84 years [2]. The combined reporting category of pneumonia and influenza remains the fifth leading cause of death in this age group [3].

Care of patients with pneumonia has been the target of quality measurement and reporting initiatives [4], [5]. Two of the largest initiatives focused on the quality of pneumonia care are the Centers for Medicare & Medicaid Service's (CMS) National Pneumonia Project and The Joint Commission ORYX® initiatives [4], [5]. Both are characterized by routine measurement of performance on process measures of care given at the time of admission (antibiotic timing and selection, oxygenation assessment, and blood cultures) and before discharge (smoking cessation counseling, and provision of influenza and pneumococcal polysaccharide vaccines).

Although measuring processes of care for pneumonia has accelerated the pace of quality improvement [6]–[8], there is an ongoing need to develop new measures of pneumonia quality that focus on patient outcomes, care transitions, and efficiency of care. Process measures can provide important information about the quality of care and guide improvement efforts; however, these measures necessarily focus on a small spectrum of the overall care provided to hospitalized patients. Outcome measures that reflect the patient's subsequent health status, such as satisfaction with care, functional status, morbidity, mortality, or parameters such as length of stay or costs of care, are generally regarded as the indicators that are most meaningful to patients and/or payers [6], [9], [10]. CMS has begun focusing resources of the development of new measures of patient outcomes, in part because of provisions in Section 5001 of the Deficit Reduction Act (DRA) of 2005 (Public Law 109–171) that require the Secretary of Health and Human Services to begin reporting hospital quality measures of process, structure, outcome, patients' perspective on care, efficiency, and costs of care.

This report describes the 30-day risk-standardized mortality model based on Medicare claims for patients discharged with a diagnosis of pneumonia developed for public reporting. This effort is an extension of an ongoing initiative by CMS to develop outcomes measures for common conditions that are based on available national data. In the first phase of the CMS initiative, risk-standardized measures of 30-day mortality for acute myocardial infarction [11] and heart failure [12] were developed using administrative data and were demonstrated to have properties suitable for public reporting [13]. CMS reports annually the measure results for all 3 conditions for the nation's acute care hospitals.

## Methods

### Patient Sample

The target population was patients aged 66 years and older who were hospitalized in non-governmental, acute care hospitals with a discharge diagnosis of pneumonia and who were enrolled in fee-for-service Medicare coverage in the 12 months before and including the time of their index hospitalization. A patient was excluded if there was incomplete administrative data for the period 12 months before the index admission date; if the patient was not enrolled in Medicare for the entire year before admission or if the patient was enrolled in a managed care (Medicare+Choice) plan during the previous year; if pneumonia was listed as a secondary diagnosis (to eliminate pneumonia resulting from complications of hospitalization); and if the patient was discharged alive and not against medical advice within the first day of admission because of concerns about the accuracy of the pneumonia diagnosis. For patients with more than 1 hospitalization, only 1 admission was randomly selected. We did this to ensure the independence of the observations. Thus our sample included all patients having a pneumonia diagnosis during a particular year.

For patients who were transferred from one institution to another, we linked the hospitalizations into an episode of care. The hospitalization was considered to have begun on admission to the initial hospital. A patient needed to be admitted as an inpatient at both the transferring and receiving hospital, and was required to have a pneumonia discharge diagnosis at both hospitals. To exclude admissions of patients discharged and subsequently readmitted, transfers must have occurred within 1 day or less, counting from the discharge date of the initial hospitalization. For the purposes of risk-adjustment, comorbid (“pre-existing”) conditions were identified from the initial (index) admission only to avoid designating index admission-related complications as pre-existing conditions in the receiving hospital. Comorbid conditions that may have represented complications of the index admission were not included in the risk-adjustment model. For the purposes of calculation of risk-stratified mortality rates, the initial hospital was designated as the responsible institution for the patient's episode of care.

### Derivation and Validation Cohorts

**Figure 1:**
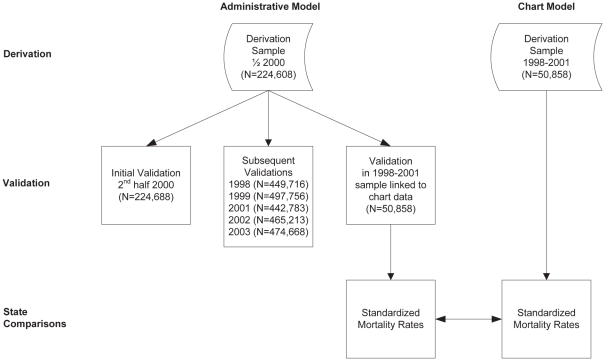
Development of the derivation and validation cohorts for the pneumonia mortality models. The administrative derivation cohort included a stratified random sample of half of the eligible pneumonia discharges for calendar year 2000. Subsequent administrative validation cohorts included the other half of eligible discharges from 2000, and all eligible discharges for calendar years 1998, 1999, and 2001–2003. The chart model derivation cohort included 50,858 eligible patients with abstracted medical record data. An additional administrative validation cohort using the claims of patients in the chart model derivation cohort was created to compare state-specific standardized mortality rates generated by the 2 models.

We created the derivation cohort using the 2000 Medicare Provider Analysis and Review (MEDPAR) files, by selecting hospitalizations for pneumonia (*International Classification of Diseases, 9^th^ Revision, Clinical Modification* (*ICD-9-CM*) codes 481, 482.XX, 483.X, 485, 486, and 487.0. Cases were stratified by hospital and then 50% of the patients from each hospital were randomly sampled in order to include cases from all hospitals where possible (Figure 1).

We evaluated the performance of the administrative claims model in validation cohorts using the other half of the 2000 MEDPAR data, and all claims data for each of years 1998, 1999, 2001, 2002, and 2003 (Figure 1). For each year we developed the study sample using the same approach as that employed for the derivation cohort.

To compare the results of the statistical models developed from Medicare claims with those from models using data collected from medical records, we identified a sample of patients for whom we had both administrative claims and detailed chart abstracted medical record data. The medical record data were obtained from the CMS National Pneumonia Project, in which patient charts from hospitalizations for pneumonia are routinely abstracted [14]. Details of chart selection and abstraction in the National Pneumonia Project have been previously published [14]. Medical records for 2 large pneumonia cohorts of up to 850 randomly selected patients per state during each of 2 time periods between July 1998 through March 1999 or July 2000 and June 2001 were available for this study. Because the sample of abstracted medical records was not sufficient for hospital-level comparisons of the 2 models, comparisons of model performance were made at the state level.

### Outcome

The patient outcome was all-cause 30-day mortality from the time of admission. We obtained mortality information from the Medicare enrollment files by linking unique patient identifiers. Because our focus was on the hospital, our primary outcome was hospital-specific, risk-standardized mortality rates (RSMRs), defined as the hospital-predicted rate divided by the hospital-expected rate and then multiplied by the observed national rate.

### Model Derivation: Patient Predictors of Mortality

We used the *ICD-9-CM* codes to identify candidate variables for the Medicare claims model. The MEDPAR claims have data on each hospitalization for fee-for-service Medicare enrollees and include demographic information, principal and secondary diagnosis codes, and procedure codes. Diagnosis codes for comorbidities were also collected from physician and hospital outpatient files. Because there are more than 15,000 *ICD-9-CM* codes, we used the Hierarchical Condition Categories (HCC) to assemble clinically coherent codes into candidate variables [15]. This system, which includes 189 categories, was developed by physician and statistical consultants under a contract to CMS and is publicly available. The HCC candidate variables considered for this model were derived from the secondary diagnosis and procedure codes from the index hospitalization and from the principal and secondary diagnosis codes from hospitalizations, institutional outpatient visits, and physician encounters within the 12 months before the index hospitalization. We combined categories of HCC variables based on clinical judgment and bivariate associations, and eliminated candidate variables with a <1% frequency. Additional candidate variables included demographic (age, sex) and procedural factors (history of bypass surgery or percutaneous coronary intervention in the past year). A total of 138 HCC variables met the above criteria and were included in the initial model. The final number of variables in the model included 2 demographic variables (age and sex), 21 comorbidity variables, and 8 pneumonia-specific variables.

#### Model Development

We estimated hierarchical generalized linear models (HGLMs) [16]–[18]. We modeled the log-odds of mortality within 30-days of admission as a function of patient demographic and clinical characteristics, and a random hospital-specific effect. This strategy accounts for within-hospital correlation of the observed outcomes, and reflects the assumption that underlying differences in quality among the hospitals being evaluated lead to systematic differences in outcomes.

We selected the covariates for the final claims model using a backwards elimination procedure through the generalized linear model (GLM) with a logit link function approach. Because of the large number of patient observations, we chose an exit criterion for a variable with a p>0.01. For each model, we calculated several indices for assessing model performance [19] at the patient level: the area under the Receiver Operating Characteristic (ROC) curve, explained variation as measured by the generalized *R* ^2^ statistic, over-fitting indices (intercepts and slopes), and the observed outcomes in strata defined by the lowest and highest deciles based on predictive probabilities. Models with ROC areas close to 1, high *R* ^2^, over-fitting intercepts near 0 and intercepts close to 1, and a large range of observed mortality between the 2 deciles are desirable [19]. We further assessed model fit through examination of Pearson residuals. After identifying covariates for the model, we re-estimated the regression coefficients of the covariates identified from our backward elimination strategy using HGLM.

### Model Validation

#### Medicare Claims Data

We validated the model by comparing its performance in the derivation set with various validation cohorts, as described above. In each validation dataset we recalibrated the model, so that we used the same variables but fit the model to the data for each specific cohort.

#### Medical Record Data

We compared the results from the claims model with those from a model based on information abstracted from the medical record. Risk factors identified by the Pneumonia Patient Outcomes Research Team (PORT) were used as independent variables in the medical record model [20]. Because some covariates could be missing for patients based on chart review, we added a category for missing values where applicable. For each abstracted variable with missing values, we first assessed the prevalence of the missing values. We didn't include in the model any variable with a high missing value rate (greater than 10%). We then created a dummy variable to represent the missing value corresponding to the abstracted variable, and used the dummy variable together with the abstracted variable in the model. This method of modeling missing data assumes that the data are missing at random and permits inclusion of all available cases, although it is not as efficient as multiple imputation procedures. We computed measures of model fit and discrimination for the medical record model similar to those computed for the claims-based models.

### RSMRs

We calculated the RSMR for each hospital using the estimated hospital-specific parameters from the respective hierarchical models. These rates are obtained as the ratio of predicted to expected mortality (similar to the observed/expected ratio), multiplied by the national unadjusted rate [21]. The ratio is predicted mortality in each hospital, given its patient mix and hospital-specific effect, divided by the expected mortality in that hospital given the same patient mix and the national average [21]. The predicted hospital outcome is the number of expected mortalities in the “specific” hospital. Operationally, we estimated all the model parameters by regressing the outcomes on the predictors for the entire sample. We then multiplied the estimated regression coefficients by the patient characteristics observed in each hospital, added the hospital-specific estimated intercept, transformed to the probability scale, and then summed over all patients in the hospital. The expected outcome for each hospital is the number of 30-day deaths expected in the hospital given the observed case-mix. We then multiplied the, estimated regression coefficients by the patient characteristics observed in each hospital, added the national intercept, transformed to the probability scale, and then summed overall all patients in the hospital. This strategy is a form of indirect standardization. The higher a hospital's predicted 30-day mortality rate, relative to expected mortality for the hospital's particular case mix of patients, the higher its adjusted mortality rate will be.

To assess the validity of the administrative data model, we calculated state-specific RSMR using the procedures described above and compared these rates with state-specific RSMRs obtained from the medical record model. Because the selection of cases for chart abstraction as a part of the National Pneumonia Project [14] was done at the state level, the number of patients with available medical record data from each hospital was relatively small. We thus used 2 approaches to examine the relationship between the RSMRs obtained from administrative data and medical record data. First, we estimated a linear regression equation describing the linear association between the 2 rates, weighting each state by the number of hospitalizations, and calculated the intercept and the slope of this equation. A slope close to 1 and an intercept close to 0 would indicate a strong linear relationship. Second, for each state we calculated the difference between the RSMR based on the claims data and the medical record data, and then summarized the distribution of these differences among the hospitals using the average, median, and maximum difference. Differences close to 0 would support agreement between the claims-based and medical record-based approaches.

### Stability of the Model over Time

We compared the performance of the claims model over time in various validation cohorts, calculating similar model fit indices as those used in the derivation and validation samples. To assess whether we included too many risk factors in our final model, we calculated indices that quantify overfitting. Specifically, we used the coefficients estimated from the derivation model to predict the log-odds of mortality in the validation cohorts. This was accomplished by multiplying the observed risk factors in each validation cohort and summing over the covariates for a subject to obtain a mortality score. Using these scores for each subject, we then estimated a logistic regression model in which the outcome was observed mortality and the single covariate was the risk score. The intercept and slope obtained from this model are referred to as overfitting indices. If there is overfitting, we would expect the slopes to be different from 1 and the intercepts to be different from 0. We repeated this process for each validation dataset, each time calculating a risk score using the regression estimates from our derivation model.

After computing the overfitting statistics, in each validation dataset, we recalibrated the model so that we used the same variables but fit the model to the data for each specific cohort. For each model, we calculated the same indices for assessing model performance [19] as in the derivation model.

All analyses were conducted using SAS version 8.02 (SAS Institute Inc., Cary, NC). Models were fitted separately to each year of data. The hierarchical models were estimated with the use of the GLIMMIX macro in SAS. Ethics committee approval and written consent from patients for the analysis were not required. This analysis was performed under a CMS-directed contract with the Colorado Foundation for Medical Care (CFMC), the Colorado Quality Improvement Organization. As such, CFMC functions as a health oversight agency; institutional ethics committee approval and written consent for release of information is therefore not required.

## Results

### Patient Characteristics and Administrative Model

The patient study samples used to create the derivation and validation cohorts for the administrative claims models are summarized in Table S1 and Figure 1. The 2000 MEDPAR dataset included 683,280 pneumonia discharges from 4,684 hospitals. The model derivation cohort consisted of approximately half of the eligible patients discharged in 2000 resulting in 224,608 cases randomly selected from 4,644 hospitals. The mean age of the cohort was 80.2±8 years. The cohort included 55.8% women and 12.0% nonwhite patients. The overall unadjusted 30-day mortality rate for the derivation cohort was 15.1%. The median annual number of Medicare pneumonia hospitalizations from the derivation sample hospitals was 37, ranging from 1 to 396, and across these hospitals (25^th^ and 75^th^ percentiles, 18 and 67, respectively).

**Figure 2:**
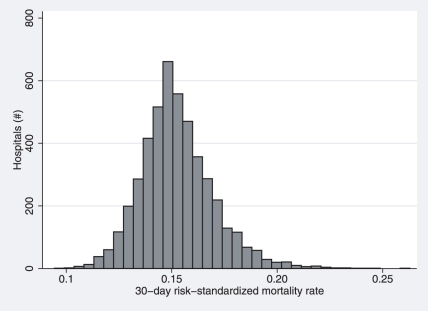
Distribution of hospital-level standardized 30-day pneumonia mortality rates. Risk-standardized mortality rates for the 4,684 hospitals are based on the administrative claims model using data for 449,296 Medicare patients discharged during calendar year 2000.

On the basis of clinical review of candidate variables, bivariate analysis, and the stepwise GLM procedure, we identified 31 variables (2 demographic and 29 clinical) for the final model. The estimated parameters from the HGLM are summarized in Table S2. No variable has a large standard error (SE) (all <0.06), and no variable has a variance inflation factor (VIF) >1.30 (VIF customarily should not exceed 10), indicating that the model does not have a collinearity problem. Model discrimination, calibration, and fit are summarized in Table S3. The p value of the goodness-of-fit for the GLM was <0.001 but the overfitting indices suggest that there is not an issue with overfitting. The area under the ROC curve was 0.72. The patient-level observed mortality rate ranges from 2.7% in the lowest predicted decile to 35.6% in the highest predicted decile, a range of 32.9%. The adjusted *R* ^2^ was 0.13. Distribution of the standardized 30-day mortality rate is shown in Figure 2. Using the complete 2000 dataset, the observed mortality rate ranged from 0% to 100% across the 4,684 hospitals. The 25^th^, 50^th^, and 75^th^ percentiles for unadjusted mortality were 14.5%, 17.3%, and 20.7%, respectively. The distribution of RSMRs, however, ranged from 13.0% to 23.7%, with 25^th^, 50^th^, and 75^th^ percentiles of 16.5%, 17.4%, and 18.3%, respectively.

### Medical Record Validation

From a total of 75,616 cases for which administrative claims and medical record abstracted data were available, the final medical record derivation sample included 50,858 cases that met inclusion criteria (Table S1). The unadjusted 30-day mortality rate for this cohort was 14.5%. Twenty covariates based on the Pneumonia PORT risk factors were included in the final model (Table S4). The area under the ROC curve was 0.77 (Table S3). The observed mortality rate ranged from 1.9% in the lowest predicted decile to 45.9% in the highest. As expected, the explained variation was higher in the medical record model (*R* ^2^ = 0.20) than in the administrative model.

In this cohort of patients with medical record data, the administrative model had an area under the ROC curve of 0.71, an observed mortality rate ranging from 2.4% in the lowest decile to 36.9% in the highest predicted decile, and an adjusted *R* ^2^ of 0.12 (Table S3).

**Figure 3:**
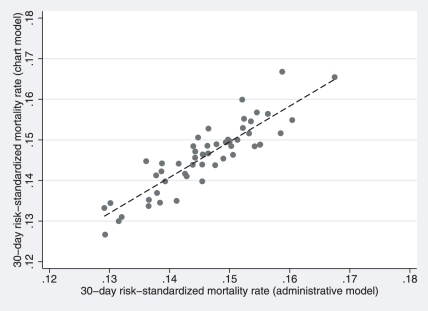
Comparison of the state-level risk-standardized mortality rates generated by the medical record model and the administrative model. Risk-standardized mortality rates were generated with both models for the 50,858 patients in the chart model derivation cohort. The correlation coefficient for rates generated by the 2 models is 0.86 (Standard Error = 0.032).

Estimated state-specific RSMRs derived from the medical record and administrative models are displayed in Figure 3. The slope of the weighted regression line of the state-specific RSMR estimated from the 2 models is 0.77 (SE = 0.06) with an intercept of close to 0 (intercept = 0.03; SE = 0.01) and a correlation coefficient of 0.86 (SE = 0.03). The mean difference in RSMR between the models is <0.0001; the range was −0.0089 to 0.0088 so that both positive and negative differences were observed.

### Stability of the Administrative Model over Time

The administrative model demonstrated good stability over time. In each of the administrative validation cohorts, the model fit was similar to the derivation cohort (Table S3). Unadjusted mortality ranged from 14.0% in 2003 to 15.0% in the 2001 dataset. The percent explained variation ranged from 0.12 to 0.13, while the area under the ROC curve was the same as in the derivation sample (0.72).

## Discussion

We have demonstrated that an administrative claims-based model for calculating 30-day pneumonia mortality rates for Medicare fee-for-service patients results in risk-standardized estimates at the state level that are highly correlated with the estimates obtained from a medical record model. For the purposes of profiling outcomes, the claims model was a very good surrogate for the medical record model, and the model was very stable over time. While insufficient samples of medical record data prevented us from validating our administrative claims model against medical record data at the level of the individual hospital, the previously developed risk-stratified measure of 30-day mortality for acute myocardial infarction, which was developed using the same methodology, was validated at the hospital level and demonstrated similar findings from the administrative and medical record models [11]. Our approach to the development of this administrative claims model for profiling hospital mortality rates is consistent with the 7 preferred attributes identified in the American Heart Association Scientific Statement that defined standards for statistical models used for public reporting of health outcomes [13].

Several aspects of the development of this model warrant additional discussion. We explicitly defined the population of patients that are eligible for inclusion in the claims model and chose the *ICD-9-CM* codes that are consistent with those used in the National Pneumonia Project for patients with a principal diagnosis of pneumonia [4]. We specifically did not include in the sample those patients with a principal diagnosis of sepsis (038.xx) or respiratory failure (518.81 or 518.84) with a secondary diagnosis of pneumonia to avoid including cases where pneumonia could have been a complication of the hospitalization. Although this is not consistent with the denominator population of the National Pneumonia Project, a medical record data element is used in the chart abstraction model to determine if pneumonia was present on admission; this was not feasible based on claims. In addition, we did not try to distinguish between community-acquired and healthcare-associated pneumonia in our model, which is also consistent with the denominator population of the National Pneumonia Project, and consistent with the work of the Pneumonia PORT and a number of studies linking processes of care to patient mortality [14], [20], [22], [23].

We chose 30 days after admission as the standard period for outcome assessment. This approach is consistent with previously National Quality Forum-endorsed mortality measures [11], [12] and the time frame of assessment used by the Pneumonia PORT, as well as with prior studies of pneumonia processes and outcomes [14], [20], [22], [23]. Evaluating mortality at 30 days (as opposed to evaluating in-hospital mortality) eliminates bias that might have occurred due to varying lengths of inpatient stay, and removes any incentive that might occur to discharge a patient from the hospital too early. A period of outcomes evaluation that is longer than the usual length of stay ensures that events early after discharge are captured, and places a premium on appropriate discharge planning [9].

We employed hierarchical modeling in the development of this model that accounts for clustering of patients within hospitals and permits separation of within- and between-hospital variation in observed outcomes. Use of hierarchical modeling reduces the chance that a hospital will falsely be characterized as an “outlier” for pneumonia mortality. For hospitals with low patient volume, predicted RSMRs will be at or near the national average.

For those patients who were transferred from one hospital to another, we assigned responsibility for patient outcomes to the first hospital. This approach increases accountability for the index hospital to appropriately make decisions about transfer of patients, and avoids hospitals that receive transfer patients who are seriously ill from being inappropriately “penalized” for having higher mortality rates [13]. Finally, all details of the development and methodology used to generate this measure of hospital 30-day mortality for pneumonia are in the public domain and subject to ongoing critique and revision.

The coefficients in our model were consistent with clinical expectations. Two exceptions were chronic obstructive pulmonary disease (HCC 108) and asthma (HCC 110), which among those patients with pneumonia were prognostically favorable. However, these results are consistent with what was found in the Pneumonia PORT. On an empirical basis, this appears to be a true relationship. The underlying mechanism is unknown, but it may be related to unmeasured factors related to pneumonia severity in these patients.

There are several issues to consider about our methodology. The model is specific to Medicare fee-for-service patients and may not be generalizable to other data sources and patient populations. However, while the model was limited to Medicare patients, approximately two-thirds of all hospitalizations for pneumonia occur in this age group. Additionally, the only national data available on which to calculate the RSMRs are Medicare claims. The need to evaluate paid hospital claims and all claims from the year before the index hospitalization results in significant time lags between patient care and reported measure rates. Consistent with measure criteria from the National Quality Forum, the mortality model does not adjust for socioeconomic status, race, or ethnicity because risk-adjusting for socioeconomic status would hold hospitals with a large proportion of such patients to a different standard of care than hospitals treating a larger proportion of patients of higher socioeconomic status [24]. An additional issue is whether an area under the ROC curve of 0.70 for discriminating survivors from non-survivors is good enough to publicly report hospital mortality. The goal of the model is to produce estimates of hospital performance. The estimates from this model agree strongly with the estimates from a medical record model with a higher C-statistic. Moreover, interval estimates of RSMRs can be obtained using the bootstrap resampling method [25], [26]. We believe that much of the unexplained variation in the RSMR derives from the latent variable of hospital quality. Finally, previous research has demonstrated varying degrees of accuracy in pneumonia coding by hospitals [27], [28].

We developed an administrative claims-based model for profiling hospitals for pneumonia mortality that is a good proxy for results from a medical record model. Despite limitations of currently available data, this model represents a valuable tool in assessing the outcomes achieved by states and hospitals in caring for patients with pneumonia, and has been endorsed by the National Quality Forum as a measure for acute care hospital performance [29]. Since initial development, several minor changes have been made to the model including expanding the cohort to include patients with viral pneumonia (adenovirus [480.0], respiratory syncytial virus [480.1], and parainfluenza virus [480.2]), and excluding patients who had enrolled in the Medicare hospice benefit before hospitalization (<1% of patients with pneumonia) [30]. CMS first began using the model for public reporting in August 2008 and hospital-specific findings are now reported on *Hospital Compare* [31].

## Supporting Information

Table S1 Pneumonia study sample used for the derivation and validation cohorts.
(DOC)

Table S2 Covariates from Hierarchical Generalized Linear Model (HGLM) included in the final administrative claims model to predict 30-day mortality.
(DOC)

Table S3 Pneumonia administrative model and medical record model performance.
(DOC)

Table S4 Covariates included in the final medical records model (HGLM).
(DOC)
