# Supplementary material for: An Administrative Claims Model for Profiling Hospital 30-Day Mortality Rates for Pneumonia Patients
Source: PLoS One. 2011 Apr 12;6(4):e17401. doi: 10.1371/journal.pone.0017401 (PMC3075250; doi:10.1371/journal.pone.0017401)
Supplement: Table S1 — Pneumonia study sample used for the derivation and validation cohorts. (DOC) [file pone.0017401.s001.doc]

|  |  | | **Age < 65 years** | **Principal Diagnosis not Pneumonia or Viral Pneumonia*** | **Repeated Admission** | **Incomplete Information†** | **Transfer‡** | | **LOS < 1 Day§** | **N** | **%** | |
| --- | --- | --- | --- | --- | --- | --- | --- | --- | --- | --- | --- | --- |
| 1998 | 691,823 | | 9.9 | 14.8 | 11.7 | 3.5 | 1.0 | | 1.6 | 449,716 | 65.0 | |
| 1999 | 744,084 | | 10.2 | 11.5 | 11.6 | 4.0 | 1.0 | | 1.9 | 497,756 | 66.9 | |
| 2000 | 683,280 | | 10.8 | 11.8 | 11.8 | 4.3 | 1.0 | | 2.1 | 449,296 | 65.8 | |
| 2001 | 690,378 | | 11.2 | 12.1 | 12.2 | 5.3 | 1.0 | | 2.3 | 442,783 | 64.1 | |
| 2002 | 733,019 | | 11.6 | 12.8 | 12.2 | 5.3 | 1.0 | | 2.4 | 465,213 | 63.7 | |
| 2003 | 758,704 | | 12.5 | 13.8 | 12.2 | 4.5 | 1.0 | | 2.6 | 474,668 | 62.6 | |
| **Medical record sample** | | | | | | | | | | | | |
| 1998-2001 | 75,616 | 10.3 | | 10.5 | 11.6 | 3.7 | | 1.1 | 2.2 | 50,858 | | 67.3 |

**Table S1. Pneumonia study sample used for the derivation and validation cohorts.**

* Patients with principal diagnosis codes of 480.X (viral pneumonia) were excluded from the population.

† Patients with incomplete information for the 12-month, pre-index admission period were excluded from the sample.

‡ After linking the “transfer-in” hospital with the “transfer-out” (index admission) hospital to define a patient’s episode of care, the records of the “transfer-in” hospital were deleted so that the case was assigned to the index admission hospital.

§ Patients who were discharged alive and not against medical advice within the first day of admission were excluded.

LOS, length of stay; MEDPAR, Medicare Provider Analysis and Review

ure; BUN, blood urea nitrogen; CI, confidence interval; CVD, cerebrovascular disease
