# Supplementary material for: An Administrative Claims Model for Profiling Hospital 30-Day Mortality Rates for Pneumonia Patients
Source: PLoS One. 2011 Apr 12;6(4):e17401. doi: 10.1371/journal.pone.0017401 (PMC3075250; doi:10.1371/journal.pone.0017401)
Supplement: Table S2 — Covariates from Hierarchical Generalized Linear Model (HGLM) included in the final administrative claims model to predict 30-day mortality. (DOC) [file pone.0017401.s002.doc]

**Table S2. Covariates from HGLM included in the final administrative claims model to predict 30-day mortality.*†‡**

| **Variable** | **Frequency, N (%)** | | **Estimate** | **Standard Error** | **Odds Ratio** | **95% CI** |
| --- | --- | --- | --- | --- | --- | --- |
| Intercept |  | | -3.19 | 0.015 |  |  |
| **Demographic** | | | | | | |
| Age – 65, (continuous variable, mean 15.2 ± 8.0) | | 449,296 (100) | 0.05 | 0.001 | 1.05 | 1.05-1.05 |
| Male | | 197,727 (44.0) | 0.24 | 0.001 | 1.28 | 1.25-1.30 |
| **Clinical** | |  |  |  |  |  |
| History of PCI | | 5,994 (1.3) | -0.65 | 0.054 | 0.52 | 0.47-0.58 |
| History of coronary artery bypass grafting | | 22,979 (5.1) | -0.46 | 0.024 | 0.63 | 0.60-0.66 |
| History of heart failure (HCC 80) | | 163,048 (36.3) | 0.23 | 0.010 | 1.26 | 1.23-1.28 |
| History of myocardial infarction (HCC 81) | | 15,786 (3.5) | 0.22 | 0.021 | 1.25 | 1.20-1.30 |
| Unstable angina (HCC 82) | | 31,282 (7.0) | -0.13 | 0.018 | 0.87 | 0.84-0.91 |
| Chronic atherosclerosis (HCC 83, 84) | | 182,007 (40.5) | -0.06 | 0.010 | 0.95 | 0.93-0.96 |
| Valvular heart disease (HCC 86) | | 51,227 (11.4) | 0.27 | 0.013 | 1.30 | 1.27-1.34 |
| Hypertension (HCC 89, 91) | | 195,898 (43.6) | -0.22 | 0.009 | 0.81 | 0.79-0.82 |
| Stroke (HCC 95, 96) | | 51,892 (11.6) | 0.14 | 0.013 | 1.14 | 1.12-1.17 |
| Cerebrovascular disease (HCC 97,98,99,103) | | 61,234 (13.6) | -0.06 | 0.012 | 0.94 | 0.92-0.96 |
| Renal failure (HCC 131) | | 34,986 (7.8) | 0.27 | 0.015 | 1.30 | 1.27-1.34 |
| COPD (HCC 108) | | 216,375 (48.2) | -0.04 | 0.009 | 0.97 | 0.95-0.98 |
| Pneumonia (HCC 111-113) | | 167,559 (37.3) | 0.11 | 0.009 | 1.11 | 1.09-1.13 |
| Protein calorie malnutrition (HCC 21) | | 37,114 (8.3) | 0.76 | 0.013 | 2.14 | 2.08-2.19 |
| Dementia (HCC 49, 50) | | 111,248 (24.8) | 0.51 | 0.010 | 1.66 | 1.63-1.69 |
| Hemiplegia, paraplegia, paralysis, functional disability (HCC 100-102, 68, 69, 177, 178) | | 34,362 (7.7) | 0.25 | 0.015 | 1.28 | 1.24-1.32 |
| Peripheral vascular disease (HCC 104, 105) | | 95,064 (21.2) | 0.12 | 0.010 | 1.13 | 1.11-1.15 |
| Metastatic or other major cancers (HCC 7-9) | | 34,385 (7.7) | 1.10 | 0.014 | 3.01 | 2.93-3.09 |
| Trauma in last year (HCC 154-156, 158-162) | | 131,100 (29.2) | 0.09 | 0.009 | 1.09 | 1.08-1.12 |
| Major psychiatric disorders (HCC 54-56) | | 44,910 (10.0) | 0.13 | 0.013 | 1.14 | 1.11-1.17 |
| Chronic liver disease (HCC 25-27) | | 5,302 (1.2) | 0.47 | 0.034 | 1.61 | 1.50-1.72 |
| Severe hematological disorders (HCC 44) | | 12,878 (2.9) | 0.28 | 0.022 | 1.32 | 1.26-1.38 |
| Iron deficiency and other/unspecified anemias and blood disease (HCC 47) | | 145,841 (32.5) | 0.06 | 0.009 | 1.06 | 1.04-1.08 |
| Depression (HCC 58) | | 32,945 (7.3) | 0.11 | 0.015 | 1.12 | 1.09-1.16 |
| Parkinson's and Huntington's diseases (HCC 73) | | 19,527 (4.4) | 0.19 | 0.019 | 1.21 | 1.17-1.26 |
| Seizure disorders and convulsions (HCC 74) | | 25,163 (5.6) | 0.11 | 0.017 | 1.11 | 1.08-1.15 |
| Fibrosis of lung and other chronic lung disorders (HCC 109) | | 34,477 (7.7) | 0.10 | 0.015 | 1.11 | 1.08-1.14 |
| Asthma (HCC 110) | | 21,780 (4.9) | -0.53 | 0.025 | 0.59 | 0.56-0.62 |
| Vertebral fractures (HCC 157) | | 14,602 (3.3) | 0.20 | 0.021 | 1.22 | 1.17-1.27 |

* Based on the 2000 derivation and validation combined cohort consisting of 449,296 cases randomly selected from 4,684 hospitals.

† HCC are used to assemble clinically coherent ICD-9-CM codes into single variables [15]. All HCC variables in the model represent “or” conditions from all data sources (hospital inpatient principal or secondary diagnoses, hospital outpatient, and clinician data).

‡ All variables are statistically significant (p < 0.001). Estimate of between-hospital variance = 0.059 (SE = 0.003).

CI, confidence interval; COPD, chronic obstructive pulmonary disease; HCC, hierarchical condition categories; HGLM, hierarchical generalized linear model; ICD-9-CM, International Classification of Diseases, Ninth Revision, Clinical Modification; PCI, percutaneous coronary intervention; SE, standard error
