# Supplementary material for: An Administrative Claims Model for Profiling Hospital 30-Day Mortality Rates for Pneumonia Patients
Source: PLoS One. 2011 Apr 12;6(4):e17401. doi: 10.1371/journal.pone.0017401 (PMC3075250; doi:10.1371/journal.pone.0017401)
Supplement: Table S3 — Pneumonia administrative model and medical record model performance. (DOC) [file pone.0017401.s003.doc]

**Table S3. Pneumonia administrative model and medical record model performance.**

| **Model** | **Overfitting Indices (Intercept, Slope)** | **Adjusted *R*^2^*** | **Discrimination Predictive Ability† (lowest decile, highest decile)** | **ROC Curve Area** |
| --- | --- | --- | --- | --- |
| **Administrative derivation sample** |  |  |  |  |
| 2000 (N = 224,608) | (0, 1) | 0.13 | 2.7%-35.6% | 0.72 |
| **Administrative validation samples** |  |  |  |  |
| 2000 (N = 224,688) | (0.002, 0.999) | 0.13 | 2.7%-35.4% | 0.72 |
| 1998 (N = 449,716) | (0.011, 0.992) | 0.12 | 2.7%-33.5% | 0.72 |
| 1999 (N = 497,756 | (0.001, 1.001) | 0.13 | 2.6%-34.9% | 0.72 |
| 2001 (N = 442,783) | (0.004, 1.000) | 0.13 | 2.6%-34.7% | 0.72 |
| 2002 (N = 465,213) | (0.001, 0.998) | 0.13 | 2.7%-35.2% | 0.72 |
| 2003 (N = 474,668) | (0.002, 1.002) | 0.13 | 2.6%-33.8% | 0.72 |
| **Medical record sample** | | | | |
| 1998-2001 (N = 50,858) | (0, 1) | 0.20 | 1.9%-45.9% | 0.77 |
| **Linked administrative sample** | | | | |
| 1998-2001 (N = 50,858) | (0.003, 0.993) | 0.12 | 2.4%-36.9% | 0.71 |

* Max-rescaled *R*^2^; † Observed mortality rates

ROC, receiver operating characteristic
