# Supplementary material for: An Administrative Claims Model for Profiling Hospital 30-Day Mortality Rates for Pneumonia Patients
Source: PLoS One. 2011 Apr 12;6(4):e17401. doi: 10.1371/journal.pone.0017401 (PMC3075250; doi:10.1371/journal.pone.0017401)
Supplement: Table S4 — Covariates included in the final medical records model (HGLM). (DOC) [file pone.0017401.s004.doc]

**Table S4. Covariates included in the final medical records model (HGLM).***

| **Variable** | **Frequency, N (%)** | **Estimate** | **Standard Error** | **Odds Ratio** | **95% CI** |
| --- | --- | --- | --- | --- | --- |
| Intercept |  | -5.63 | 0.148 |  |  |
| Sex (female) | 27,763 (54.6) | -0.25 | 0.027 | 0.78 | 0.74-0.82 |
| Age (continuous variable) | 50,858 (100) | 0.03 | 0.002 | 1.03 | 1.03-1.04 |
| Nursing home resident | 9,376 (18.4) | 0.79 | 0.033 | 2.21 | 2.07-2.35 |
| Neoplastic disease | 3,587 (7.1) | 0.96 | 0.046 | 2.60 | 2.38-2.85 |
| Liver disease | 547 (1.1) | 0.37 | 0.117 | 1.45 | 1.15-1.82 |
| History of heart failure | 17,932 (35.3) | 0.24 | 0.028 | 1.27 | 1.20-1.34 |
| History of CVD | 9,984 (19.6) | 0.10 | 0.033 | 1.10 | 1.04-1.18 |
| History of renal disease | 4,475 (8.8) | -0.05 | 0.045 | 0.95 | 0.87-1.04 |
| Altered mental status | 9,888 (19.4) | 0.60 | 0.031 | 1.81 | 1.71-1.93 |
| BUN ≥ 30 mg/dl | 12,790 (25.2) | 0.88 | 0.031 | 2.42 | 2.27-2.57 |
| Systolic BP < 90 mm Hg | 1,735 (3.4) | 0.62 | 0.059 | 1.85 | 1.65-2.08 |
| Temperature < 35°C or ≥ 40°C | 885 (1.7) | 0.42 | 0.087 | 1.52 | 1.28-1.80 |
| Pulse ≥ 125/min | 4,140 (8.1) | 0.46 | 0.045 | 1.58 | 1.45-1.73 |
| Arterial pH < 7.35 | 2,069 (4.1) | 0.82 | 0.056 | 2.26 | 2.02-2.52 |
| Respiratory rate ≥ 30/min | 8,698 (17.1) | 0.50 | 0.033 | 1.64 | 1.54-1.75 |
| Sodium < 130 mmol/liter | 2,539 (5.0) | 0.48 | 0.057 | 1.62 | 1.45-1.81 |
| Glucose ≥ 250 mg/dl | 2,862 (5.6) | 0.14 | 0.054 | 1.15 | 1.04-1.28 |
| Hematocrit < 30% | 4,080 (8.0) | 0.27 | 0.044 | 1.30 | 1.20-1.42 |
| Partial pressure arterial O_2_ <60 mm Hg | 6,318 (12.4) | 0.21 | 0.045 | 1.24 | 1.14-1.35 |
| Pleural effusion | 11,295 (22.2) | 0.39 | 0.031 | 1.47 | 1.38-1.56 |
| BUN missing | 7,182 (14.1) | 0.12 | 0.153 | 1.13 | 0.84-1.53 |
| BP missing | 5,492 (10.8) | 0.81 | 0.440 | 2.25 | 1.71-2.96 |
| Temperature missing | 5,534 (10.9) | 0.51 | 0.326 | 1.66 | 0.88-3.15 |
| Pulse missing | 5,490 (10.8) | -0.53 | 0.458 | 0.59 | 0.24-1.44 |
| Arterial pH missing | 33,964 (66.8) | -0.22 | 0.348 | 0.80 | 0.40-1.58 |
| Respiratory rate missing | 5,586 (11.0) | 0.09 | 0.233 | 1.10 | 0.70-1.73 |
| Sodium missing | 7,095 (14.0) | 0.36 | 0.146 | 1.44 | 1.08-1.91 |
| Glucose missing | 7,624 (15.0) | 0.05 | 0.112 | 1.06 | 0.85-1.32 |
| Hematocrit missing | 6,685 (13.1) | 0.22 | 0.096 | 1.25 | 1.04-1.51 |
| Partial pressure arterial O_2_ missing | 34,009 (66.9) | 0.03 | 0.349 | 1.03 | 0.52-2.03 |

*Based on the 1998-2001 medical records derivation cohort consisting of 50,858 cases. The unadjusted 30-day mortality rate for this cohort was 14.5%.

BP, blood pressure; BUN, blood urea nitrogen; CI, confidence interval; CVD, cerebrovascular disease
